# A novel quantitative targeted analysis of X-chromosome inactivation (XCI) using nanopore sequencing

**DOI:** 10.1038/s41598-023-34413-3

**Published:** 2023-08-08

**Authors:** Josefin Johansson, Sarah Lidéus, Ida Höijer, Adam Ameur, Sanna Gudmundsson, Göran Annerén, Marie-Louise Bondeson, Maria Wilbe

**Affiliations:** 1 Department of Immunology, Genetics and Pathology, Science for Life Laboratory, Uppsala University, Husargatan 3, Box 815, SE-751 08 Uppsala, Sweden; 2 Program in Medical and Population Genetics, Broad Institute of MIT and Harvard, Cambridge, MA USA; 3 Division of Genetics and Genomics, Boston Children’s Hospital, Harvard Medical School, Boston, MA USA

**Keywords:** Functional genomics, Genomics, Sequencing, Epigenetics, Gene expression, Neurodevelopmental disorders

## Abstract

X-chromosome inactivation (XCI) analyses often assist in diagnostics of X-linked traits, however accurate assessment remains challenging with current methods. We developed a novel strategy using amplification-free Cas9 enrichment and Oxford nanopore technologies sequencing called XCI-ONT, to investigate and rigorously quantify XCI in human androgen receptor gene (*AR*) and human X-linked retinitis pigmentosa 2 gene (*RP2*). XCI-ONT measures methylation over 116 CpGs in *AR* and 58 CpGs in *RP2*, and separate parental X-chromosomes without PCR bias. We show the usefulness of the XCI-ONT strategy over the PCR-based golden standard XCI technique that only investigates one or two CpGs per gene. The results highlight the limitations of using the golden standard technique when the XCI pattern is partially skewed and the advantages of XCI-ONT to rigorously quantify XCI. This study provides a universal XCI-method on DNA, which is highly valuable in clinical and research framework of X-linked traits.

## Introduction

**Figure 1:**
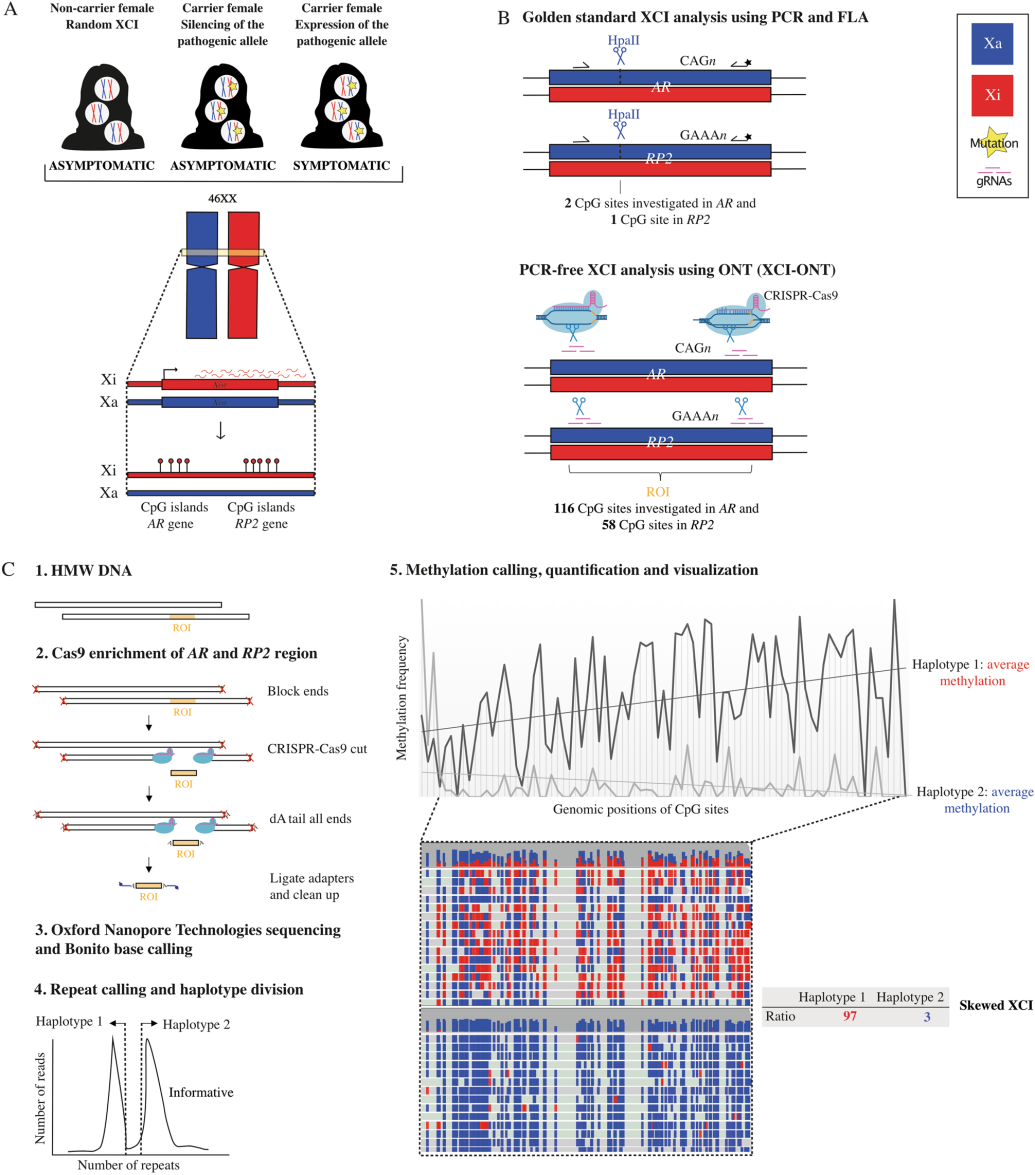
(**A**) Schematic of the X-chromosome inactivation mechanism and its effect in carrier females of X-linked traits. Blue: active X-chromosome (Xa), red: inactive X-chromosome (Xi), yellow star: pathogenic X-linked variant. Illustrations created by using adobe illustrator 2022 (available at https://adobe.com/products/illustrator). (**B**) The golden standard XCI methods uses methylation sensitive restriction enzymes (HpaII) that cuts the Xa but leaves the Xi intact. HpaII targets two and one CpG site in the Androgen receptor (*AR*) and Retinis pigmentosa 2 (*RP2*) respectively, and is followed by PCR and fragment length analysis (FLA) spanning polymorphic repetitive regions (CAG_n_ or GAAA_n_) that separates the parental alleles. Illustrations created by using Adobe Illustrator 2022 (available at https://adobe.com/products/illustrator). The XCI-ONT approach cuts the DNA independent of methylation status using CRISPR-Cas9 enrichment with three gRNAs (pink) flanking a ~ 3 kb region of interest (ROI) spanning 116 CpG sites in *AR* and 58 CpG sites in *RP2*. (**C**) XCI-ONT includes: (**1**) Dephosphorylation of 5’ ends to reduce ligation of sequencing adapters to off-target strands. (**2**) CRISPR-Cas9 system bind and cuts the ROI, and the DNA is dA-tailed for adapter ligation to Cas9 cut sides, which are both 3’ dA-tailed and 5’ phosphorylated. (**3**) The library is sequenced using Oxford Nanopore Technologies and Bonito base calling. (**4**) Calling repeats by aligning the reads to reference genomes containing all possible repeats in the regions, and the reads are divided into haplotypes by plotting the number of reads with different repeats. Lastly, methylation calling and quantification are performed using Nanopolish, and the data is visualized using Integrative Genomics Viewer (IGV). The average methylation of the ROI is calculated and the XCI ratio between the two X-chromosomes is determined.

X-chromosome inactivation (XCI) is a compensation mechanism for the difference in number of X-chromosomes between males (46XY), females (46XX) and individuals with X-aneuploidies^1^. The human molecular XCI mechanism is not completely understood^2,3^ but studies have shown that XCI is controlled by cis-acting and trans-acting factors in the X-inactivation center (Xic) including gene expression of the X-inactive specific transcript (*XIST*) and X-active specific transcript (*XACT*) genes^3^. This eventually leads to monoallelic expression, and accumulation of H3K27me3^3^ and methylation of CpG sites near promoters of genes on the inactive X-chromosome (Xi) (Fig. 1A)^4–6^. Methylation has shown to be important for the maintenance of Xi’s inactive state^7–10^. XCI in humans is initiated in the early embryonic implantation stage^3^, and the choice of which X-chromosome to be inactivated is in general described as a random process, where both X-chromosomes are equally represented^11,12^. However, preferential inactivation of one X-chromosome, so-called skewed XCI, has been observed to modify X-linked disease manifestation in carrier females, indicating that the genotype is important in the choice of active X-chromosome (Xa), possibly by cell selection due to a mutant cell disadvantage^13–16^. Firstly, preferential silencing of the pathogenic allele has been associated with a selective female survival or a less severe effect of X-linked traits. Extreme skewing has been shown to be a good indicator for the presence of pathogenic X-linked variants in carrier females^15,17,18^ and XCI analyses in family relatives therefore often assist in the interpretation of X-linked variants. Secondly, expression of the pathogenic allele may lead to manifestation of phenotypes in carrier females of X-linked traits^19–28^ and can explain phenotypic differences observed in affected carrier females and individuals with X-aneuploidies^21,24,29–36^. Of note, XCI analyses are recommended to be performed in relevant tissues, at different ages and in non-smokers^37–44^. In general, the definition of skewing has been > 80:20 but due to limitations in the golden standard technique used for XCI analysis, quantification of the skewedness has not been recommended for other than 100:0 silencing, highlighting the need of a quantitative method.

The golden standard XCI analysis uses Methylation Sensitive Restriction Enzymes (MSREs), PCR and Fragment Length Analysis (FLA) targeting two regions on the X-chromosome; the first exon of the human androgen receptor (*AR*) gene^45^ and the promoter region of the human X-linked retinitis pigmentosa 2 (*RP2*) gene^46^. Methylation of CpG sites in these regions are associated with XCI in humans^46–48^ and by using different MSREs with various target sequences, one or a few CpG sites in these genes are often investigated using the golden standard analysis. Methylation of CpGs in *AR* and *RP2* are of specific interest in XCI analyses due to their proximity to highly conserved polymorphic repetitive elements (CAG_n_ in *AR* and GAAA_n_ in *RP2,* heterozygosity rate 0.97 combined) that can be used for separating the parental alleles using FLA (Fig. 1B). However, the golden standard analysis is not robust since it relies on MSREs for digestion, is PCR-based and semi-quantitative. The results are often difficult to interpret due to PCR stutter peaks, secondary structures and/or polymorphisms in the fragment affecting the fragment size and thereby biasing the separation of alleles. In addition, PCR and FLA are not suitable for quantifying imbalanced expression of the two X-chromosomes often leaving the smaller fragment in favor^49^.

Quantification is necessary when the level of skewedness is < 100:0, and in the grey zone of the skewing definition (> 80:20), often used in the interpretation of X-linked variants. In addition, quantification can be very useful when XCI is modifying disease e.g. in the investigation of carrier females presenting symptoms due to expression of the pathogenic allele (incomplete silencing). Approaches for quantifying the levels of XCI on the maternal and paternal allele have been proposed before but this requires either bisulfite conversion and PCR^50^ or paired RNA and DNA sequencing data of the *XIST* gene using informative transcribed heterozygous single nucleotide variants (SNVs) to separate the parental alleles^41,51^. A SNV is not as informative as repeated elements and can therefore not be used to distinguish between individuals. This highlights the need for a universal quantitative XCI analysis on DNA to be used for research applications and clinical diagnostics related to X-linked traits.

Oxford nanopore technologies (ONT) sequencing can be used to detect CpG methylation revealed by changes in the raw electrical signal during the sequencing process. Long reads are powerful to investigate methylation over repetitive regions and GC-rich sequences such as CpG islands. In addition, high coverage without amplification is a very useful quantitative measurement of methylation. However, high read depth of the whole genome is expensive and therefore an enrichment approach could be advantageous. Recently, an amplification-free targeted library preparation prior to long-read sequencing was released using the CRISPR-Cas9 system to effectively sequence complicated targeted regions with high-coverage and no PCR bias^52–56^. Analysis without PCR steps are important to avoid allelic dropout and allow quantitative measurement of methylation. Here, we demonstrate a novel strategy to quantify XCI using CRISPR-Cas9 enrichment of the *AR* and *RP2* regions along with ONT sequencing for repeat and methylation detection. The novel strategy is referred to as XCI-ONT in this paper and is compared to the golden standard XCI method (Fig. 1B,C).

## Results

### XCI investigation using golden standard method of *AR* and *RP2*

In this study, XCI was first investigated using the golden standard PCR-based strategy of the *AR* and *RP2* genes. The investigated family was previously described with X-linked intellectual disability (OMIM #300966) caused by a *TAF1* variant (c.3568C > T;p.(Arg1190Cys), NM_004606.4) where carrier females had ~ 100:0 skewed XCI when investigating the *AR* gene^57^. The XCI result was confirmed in this study by investigating the *RP2* locus of two asymptomatic carrier females (IV:8, III:10) and one asymptomatic non-carrier female (III:7) (Supplementary Fig. 1). The two asymptomatic carrier females presented a ~ 100:0 skewed XCI status consistent with the maternal X-chromosome being silent (carrying the pathogenic variant). The asymptomatic non-carrier female had expression of both alleles i.e. a random XCI status, however the method presents variable result between experiments and contrasting results in the different genes (63:37 in *AR* vs. 47:53 in *RP2).* In addition, three females (female I, II and III) were investigated using the golden standard method. All individuals showed different ratios of XCI for both *AR* (female I 62:38, female II 47:53 and female III 81:19) and *RP2* (female I 81:19, female II 60:40 and female III 87:13) (Table 1, Supplementary Fig. 2).

**Table 1:** XCI status of all samples in the *AR* and *RP2* gene.

Sample	OMIM	Female disease status	XCI status	
			*AR* (FLA*/ONT)	*RP2* (FLA*/ONT)
IV:8	#300966	Carrier	100:0/95:5	100:0/91:9
III:10	#300966	Carrier	100:0/97:3	100:0/91:9
III:7	#300966	Non-carrier	63:37/31:69	47:53/34:66
Female I.I	NA	NA	62:38/28:72	81:19/26:74
Female I.II	NA	NA	NA/27:73	NA/27:73
Female II	NA	NA	47:53/54:46	60:40/67:33
Female III	NA	NA	81:19/81:19	87:13/92:8

*FLA* fragment length analysis/golden standard analysis, *ONT* Oxford nanopore technologies.

*FLA is a semi-quantitative method and measuring peak heights is not recommended for other than extreme skewedness (> 90:10).

### XCI investigation using XCI-ONT of *AR* and *RP2*

**Figure 2:**
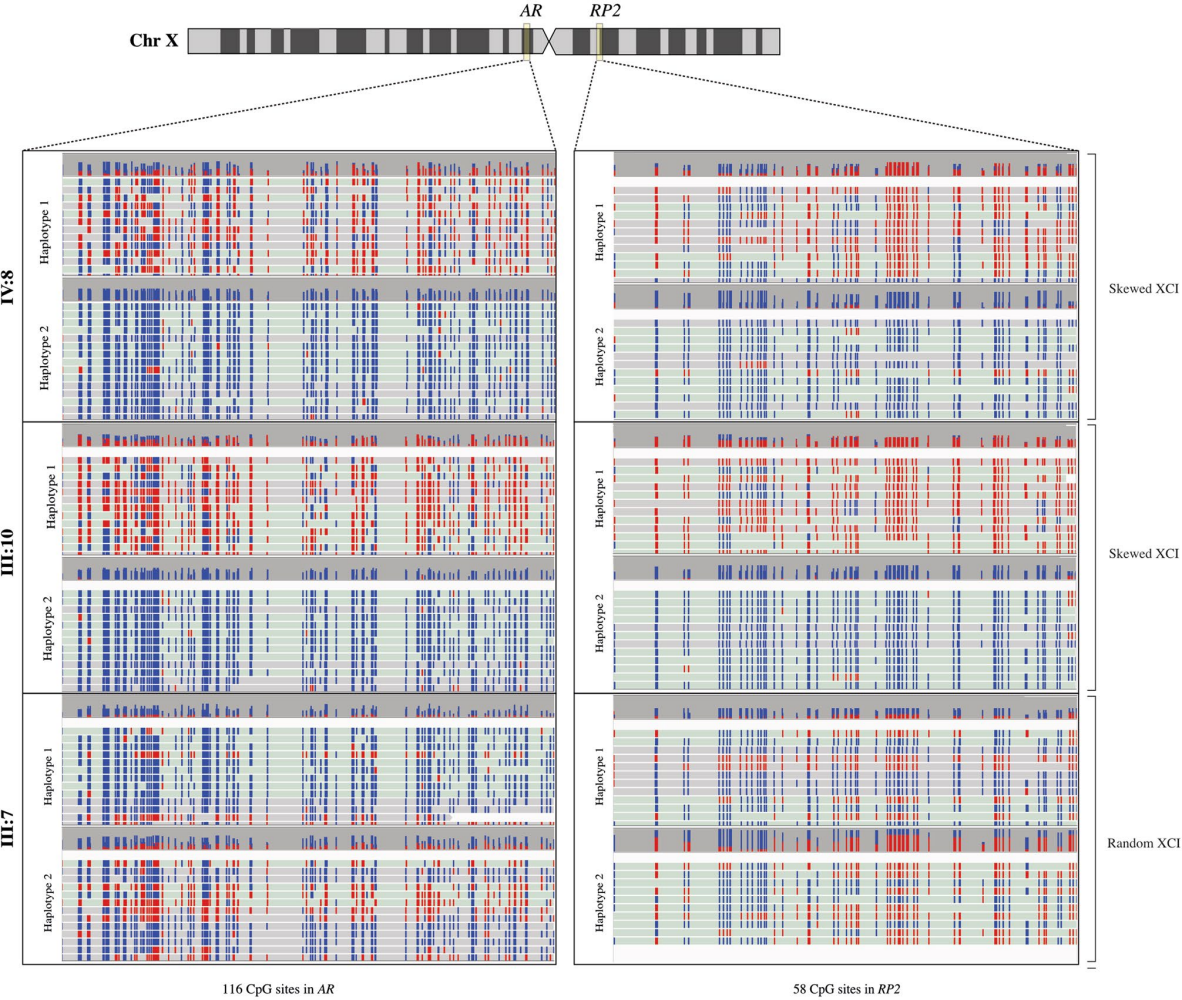
Visualization of XCI-ONT result using Integrative Genomics Viewer (IGV) presenting methylated CpG sites (red; Xi) and unmetylated CpG sites (blue; Xa) across the reads in the Androgen receptor (*AR*) and Retinis pigmentosa 2 (*RP2*) genes. Asymptomatic carrier females (IV:8, III:10) present a skewed XCI pattern and asymptomatic non-carrier females (III:7) of the same family have a random XCI status. Top bar in each haplotype visualization indicates the read coverage (height) and the percentage of methylated and unmethylated calls at each position.

To address the limitations of the golden standard technique and quantify the ratio of active maternal or paternal X-chromosomes, we developed XCI-ONT and applied it on the same samples (Fig. 1B,C). XCI-ONT uses the Cas9-enrichment protocol^53^ with three gRNAs on both sides of a ~ 3 kb region spanning the same repeats and CpG sites as the golden standard method of the *AR* and *RP2* genes (Supplementary Table 1). The region enrichments were followed by DNA sequencing and simultaneous methylation detection using ONT sequencing. This enables direct detection of repeats without PCR-bias and methylation detection of 116 CpGs in *AR* (chrX: 67543761–67546170, hg38) and 58 CpGs in *RP2* (chrX: 46836539–46837273, hg38), in comparison to the golden standard technique investigating only one or two CpGs per gene^45,46^. The XCI-ONT investigation generated between 43 and 155 reads on target of which 32–105 were used to calculate the methylation frequencies to separate the haplotypes (Supplementary Table 2). The repeats were detected by alignment to reference genome containing a range of naturally occurring repeats lengths in each gene respectively. The number of repeats in the novel XCI-ONT assay in *AR* and *RP2* was consistent with the base pair differences detected using the golden standard assay (Supplementary Figs. 1, 2 and 3). Methylation calling and calculation of the methylation frequency were performed using Nanopolish^58^. The methylation status was visualized, and the XCI status was calculated using the average methylation frequency in said regions followed by calculating the ratio of the average methylation between the alleles in each gene. The two asymptomatic carrier females (IV:8 and III:10) presented a skewed XCI status using XCI-ONT with a methylation ration of 95:5 (IV:8) and 97:3 (III:10) in *AR*, and 91:9 (IV:8) and 91:9 (III:10) in *RP2*. The asymptomatic non-carrier female (III:7) presented a random XCI status with a methylation ratio of 31:69 in *AR*, and 34:66 in *RP2* (Table 1, Fig. 2 and Supplementary Table 3). Females I and II displayed a random XCI status, and female III presented a random XCI status for *AR* but a skewed XCI status for *RP2*. Female I was analyzed two times (female I.I and female I.II) with a new DNA sample to investigate the robustness of the method. The ratios were as following; 28:72 (female I.I), 27:73 (female I.II), 54:46 (female II), and 81:19 (female III) in *AR* and 26:74, (female I.I) 27:73 (female I.II), 67:33 (female II), and 92:8 (female III) in *RP2.* The results from female I was highly consistent between the two separate runs (28:72 vs. 27:73), demonstration the robustness and specificity for XCI-ONT (Table 1, Supplementary Fig. 4 and Supplementary Table 3). The variability of methylation calls between the two haplotypes across all reads and analyzed CpG sites were investigated and visualized. A distinct pattern for the majority of CpG sites between methylated or unmethylated haplotypes can be seen for IV:8 and III:10 for both *AR* and *RP2* (Supplementary Fig. 5A–D). For females I and II the different haplotypes cannot be distinguished using methylation status (Supplementary Fig. 5E–L). Female III exhibits no distinction between the haplotypes for *AR* but shows a clear division between the haplotypes for *RP2* (Supplementary Fig. 5M,N).

## Discussion

We demonstrate a combination of CRISPR-Cas9 enrichment and ONT sequencing (XCI-ONT) as a novel successful strategy to quantify XCI in females, which can be used to investigate X-linked traits. Here, XCI was investigated in a family with X-linked intellectual disability^57^, using the golden standard PCR-based strategy of the *AR* and *RP2* genes, and then compared to the XCI-ONT result of the same genes. The golden standard XCI investigation of carrier females (IV:8 and III:10) revealed a ~ 100:0 skewed XCI status in the *AR* and *RP2*, resulting in silencing the pathogenic allele as a protective mechanism to disease. The golden standard XCI investigation of an asymptomatic non-carrier female of the same family (III:7) as well as three additional individuals (female I-III) expressed both alleles (random XCI), however the golden standard method presented variable result between experiments and contrasting results in the different genes (63:37 in *AR*, 47:53 in *RP2* for III:7 and 62:38 in *AR*, 81:19 in *RP2* for female I). The results highlight the limitations of using the golden standard method when the XCI pattern is only partially skewed^45,46^.

To address this, and quantify the ratio of active maternal or paternal X-chromosomes, we developed XCI-ONT, and applied it on the same samples as in the golden standard analysis. The XCI-ONT method detects 116 CpGs in the *AR* gene and 58 CpGs in *RP2* gene, compared to only one or two CpGs per gene using the golden standard method^45,46^. The two asymptomatic carrier females presented a skewed XCI status with 95:5 (IV:8) or 97:3 (III:10) in the *AR* gene, and 91:9 in the *RP2* gene (IV:8 and III:10), which is consistent with the golden standard results, affirming the established XCI-ONT protocol presented in this paper. The asymptomatic non-carrier female (III:7) and female I presented a random XCI status using XCI-ONT with a methylation ratio of 31:69 and 28:72 in *AR* and 34:66 and 26:74 in *RP2* respectively, implying a distinctly different and robust detection using XCI-ONT compared to the golden standard investigation that presented contrasting results between genes and experiments (63:37 and 62:38 in *AR*, 47:53 and 81:19 in *RP2*). These results display a robust XCI detection of different ratios in both genes and confirm that the quantitative XCI-ONT investigation is preferred when XCI pattern is partially skewed. Despite the small sample size of this study, the results show a more accurate XCI investigation with great quantitative potential using the novel XCI-ONT strategy compared with the semi-quantitative golden standard method. We also show that reanalyzing an individual using a new DNA sample yields the same result demonstrating the robustness of the method.

To distinguish the alleles, both the golden standard and XCI-ONT strategy uses the advantage of highly conserved polymorphic repetitive elements in *AR* and *RP2*. Repeats are useful for separating chromosomes since parental alleles often differ in number of repeat units. The golden standard investigation uses FLA for an indirect detection of the repeats and separates the alleles by fragment size. Consequently, the haplotype division can be biased by indels and fluorophores or secondary structures affecting the detected fragment length. ONT has the power of direct detection of repeats, underlining the power of using XCI-ONT for haplotype division. This conclusion was supported because the difference in repeat lengths between the two haplotypes was consistent when comparing the two methods, except in female I where the second haplotype show 22 repeats by FLA and 39 repeats using XCI-ONT. This is most likely due to the fact that FLA is a PCR based method which cannot handle the large number of repeats.

To investigate XCI skewedness, both the golden standard and XCI-ONT strategy uses the methylation level of the different haplotypes in *AR* and *RP2*. However, the golden standard method has limitations since it relies on MSREs for digestion and only investigate methylation of one or a few CpG sites in the region. In comparison, the XCI-ONT strategy investigates methylation over 116 CpG sites in *AR* and 58 CpG sites in *RP2* using ONT sequencing. To consider the methylation calling error frequency, only reads with steady methylation calls have been included in this analysis (see “Methods” section). Thus, some CpG sites may end up with no methylation status (Supplementary Fig. 5), and therefore the XCI-ONT status is reported as a ratio of the average methylation between the chromosomes. Future studies that include more samples can reveal which sites that are the most reliable to use in analysis and therefore fine-tune this method and improve accuracy even further. In theory, confirmed by the results in this study, XCI-ONT results in a more accurate XCI measurement in comparison to golden standard methods, especially with partially skewed females, indicating the power of using ONT and more than one CpG site in XCI analyses. This is confirmed by the variability of methylation seen across the CpG sites in the two haplotypes of all investigated individuals (Supplementary Fig. 5).

In this study, both the *AR* and *RP2* gene have been used to confirm the XCI status. The methylation ratio of the alleles may differ slightly in the genes due to poor haplotype division caused by ≤ 2 repeats in difference in *RP2*, more CpGs in the investigated *AR* region or methylation calling errors. XCI-ONT holds the potential of increasing the number of gRNAs targeting additional X-linked genes, improving the accuracy further. In addition, to date, a limitation in the methylation calling only allows methylation detection within 10 base pair windows and therefore XCI-ONT cannot be compared to the golden standard method at a CpG site level. However, although this is not a possibility with Nanopolish yet, this research is constantly evolving and is expected to be possible soon.

In parallel to our study, ONT sequencing was demonstrated as a promising tool to investigate XCI across the X-chromosome^6^, but ONT sequencing as a targeted approach to separate the haplotypes and quantify XCI has to our knowledge not been reported before. Moreover, the performance of ONT sequencing compared to golden standard XCI methods have not been demonstrated before.

To summarize, we demonstrate for the first time, XCI-ONT as a novel successful strategy to quantify XCI in carrier females of X-linked traits. This is useful in clinical work and research when the patient is in the grey zone of the skewing definition (> 80:20), often assisting in the interpretation of X-linked variants. In addition, quantification can be highly useful in the investigation of carrier females manifesting symptoms due to expression of the pathogenic allele. Quantification of XCI can illuminate the mechanism of disease, and provide useful information for improving prenatal risk assessment^36^, diagnostics and treatment development of X-linked traits. Lastly, we would like to illuminate the possibility that different disorders and pathogenic variants requires different levels of expression to develop symptoms and stress the use of a quantitative XCI investigation to answer these questions. A related application for XCI-ONT is monitoring pharmacological Xi reactivation, which has been suggested as treatment for X-linked disorders due to skewed XCI^59–62^.

## Methods

### Patient samples and ethical considerations

Ethical approval was received from the local ethics committee of the Swedish Ethical Review Authority for human research in Uppsala, Sweden (Dnr 2012/321). The samples were recruited at the Department of Clinical genetics, Uppsala University hospital and informed consent was received from all participants. The study was conducted according to the guidelines of the Declaration of Helsinki. HMW genomic DNA were extracted from 200 µl blood of six females using Nanobind CBB Big DNA kit (Circulomics) according to standard procedures or using the salting-out method (standard protocol available upon request) followed by clean-up with Nanobind CBB Big DNA kit (Circulomics). The family investigated in this study was previously described with X-linked intellectual disability (OMIM #300966) caused by a c.3568C > T;p.(Arg1190Cys) variant in the *TAF1* gene (NM_004606.4)^57^. DNA from two carrier females (III:10, IV:8) and one non-carrier female (III:7) as well as three unrelated females (female I-III) was included in the current analysis.

### XCI investigation using golden standard methods of the *AR* and *RP2* genes

Two hundred ng of genomic DNA were cut using methylation sensitive HpaII FastDigest in a total volume of 20 µl following manufacturer’s instructions (Thermo Fisher Scientific). PCR spanning the *AR* gene was performed using Long-range PCR kit (Qiagen) with input of 50 ng DNA or 2 µl digested DNA with primers and cycling conditions described before^45^. The *RP2* gene was amplified in a 20 µl reaction containing 1X PCR reaction buffer, 250 nM dNTPs, 1uM of each primer, 1U Taq polymerase, 50 ng DNA or 2 µl digested DNA with primers and thermal cycler conditions described before^46^. Genotyping was performed using FLA on the 3130xl ABI Genetic Analyzer with ROX500 Size Standard (Thermo Fisher Scientific). The Amplified Fragment Length Polymorphism (AFLP) was determined using Geneious Prime version 2022.01 with microsatellite plugin (https://www.geneious.com). The peak heights were measured using ImageJ software^63^ followed by calculation of the height ratio. DNA from a female sample with known XCI pattern (100:0) were used as a control throughout the analysis pipeline.

### XCI investigation using XCI-ONT of the *AR* and *RP2* genes

Three CRISPR RNAs (crRNAs) on both sides of each region of interest in the *AR* and *RP2* genes were designed using CHOPCHOP^64^ and selected according to the previously described instructions (Cas-mediated PCR-free enrichment protocol version: ENR_9084_v109_revD_04Dec2018). All custom designed Alt-R crRNAs (Integrated DNA Technologies, Supplementary Table 2) were pooled as an equimolar mix of crRNAs (100 µM) and assembled with trans-activating crRNAs (tracrRNAs) (Integrated DNA Technologies, cat 1073190) using Duplex buffer (Integrated DNA Technologies, cat 11010301) according to manufacturer’s instructions. Cas9 enrichment was performed according to manufacturer’s instructions using on 4–5 μg DNA and Long fragment buffer (Cas-mediated PCR-free enrichment protocol SQK-CS9109). Samples were run on a MinION R9.4.1 flow cell and operated using the MinKNOW software (version 20.10.3).

### Methylation analysis of Oxford nanopore data

Base calling was performed using Bonito basecaller (version 0.3.8) and reads were aligned to the human reference genome (GRCh38/hg38) using minimap2 (version 2.18-r1015). Only reads targeting the regions of interest in the *AR* (chrX:67543761–67546170, hg38) and *RP2* gene (chrX:46836539–46837273, hg38) were considered for further analysis. To enable repeat-calling, an in-house database was created with reference genomes in containing 5–40 CAG repeats (*AR* gene) and 5–30 GAAA repeats (*RP2* gene). The range of repeat numbers used in the alignment has previously been described in the unaffected general population (ranging from 9 to 38 in *AR* and 10–20 in *RP2*)^46,65^. Repeat calling was performed by alignment to the in-house database using minimap2 (version 2.18-r1015) and number of repeats in the reads were identified using Integrative Genomics Viewer (version 2.10.0, base quality > 20). The data were visualized by plotting the number of reads (y-axis) containing any repeat in the range of 5–40 repeats in *AR* or 5–30 repeats in *RP2* (x-axis) (Supplementary Fig. 3). The reads were divided into haplotypes based on the repeat count and comparison with the golden standard results. Methylation calling and calculation of the methylation frequency was performed using Nanopolish software package (version 0.12.0). Only sites with a log-likelihood ratio > 2.5 (methylated) or < − 2.5 (unmethylated) were included in the methylation analysis (Supplementary Fig. 5). Bam-files were converted using the “converting bam for igv” package^66^ and the methylation status was visualized using Integrative Genomics Viewer bisulfite mode CG (version 2.10.0). The XCI status was established by using the average methylation frequency in the chrX:67543761–67546170 region (*AR* gene, 116 CpG sites, hg38) and chrX:46836539–46837273 region (*RP2* gene, 58 CpG sites, hg38) followed by calculating the ratio of the average methylation between the alleles in each gene.

### Supplementary Information

Supplementary Figure 1.

Supplementary Figure 2.

Supplementary Figure 3.

Supplementary Figure 4.

Supplementary Figure 5.

Supplementary Table 1.

Supplementary Table 2.

Supplementary Table 3.

Supplementary Legends.

## Data Availability

The datasets generated and analyzed during the current study are available in the European Nucleotide Archive (ENA) repository, [Study Accession: PRJEB53974, ERP138789]”.
