# Supplementary figures and images for: A novel quantitative targeted analysis of X-chromosome inactivation (XCI) using nanopore sequencing
Source: Sci Rep. 2023 Aug 8;13:12856. doi: 10.1038/s41598-023-34413-3 (PMC10409790; doi:10.1038/s41598-023-34413-3)

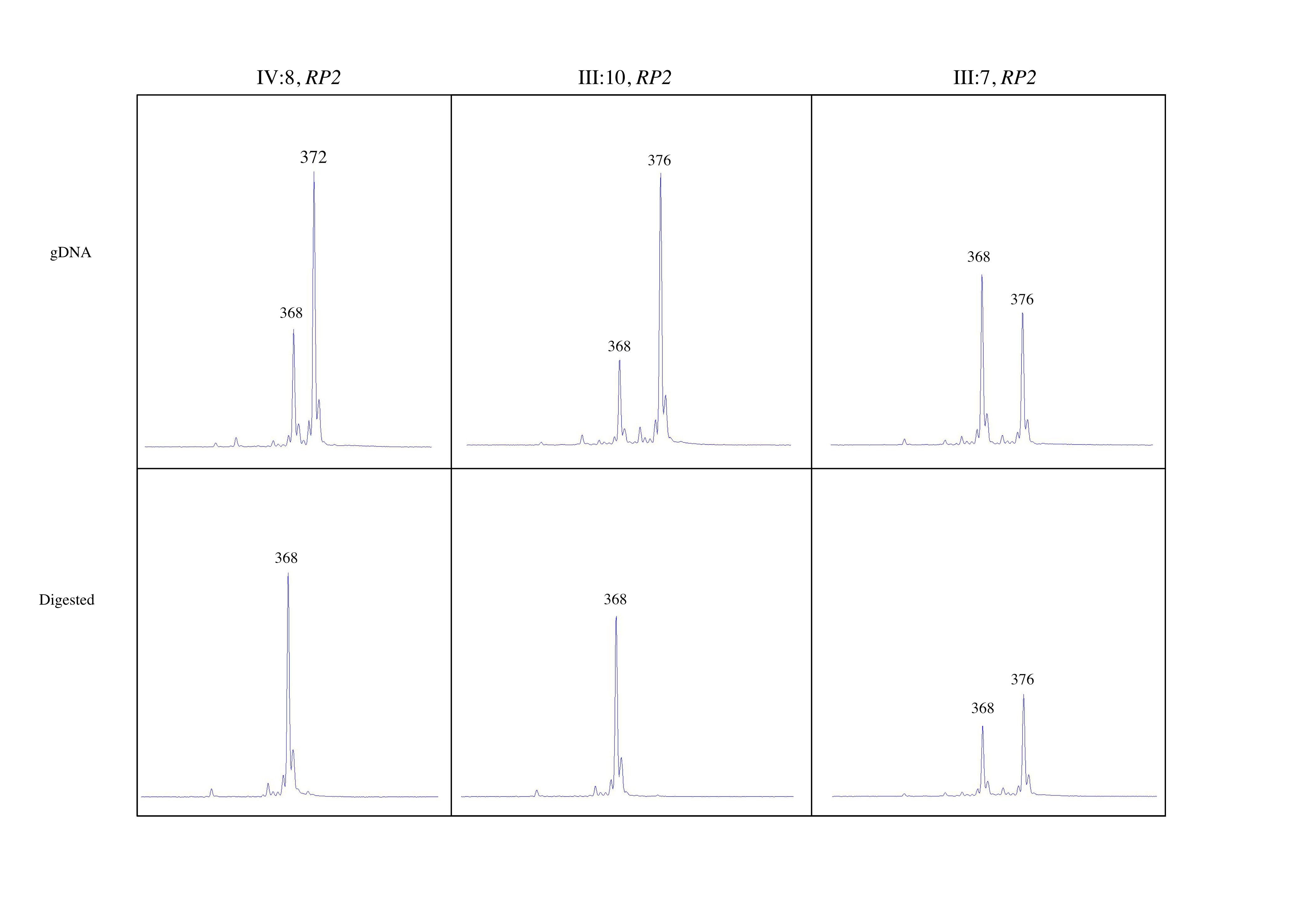

Supplement: Supplementary file 1 — Supplementary Figure 1. [file 41598_2023_34413_MOESM1_ESM.jpg]

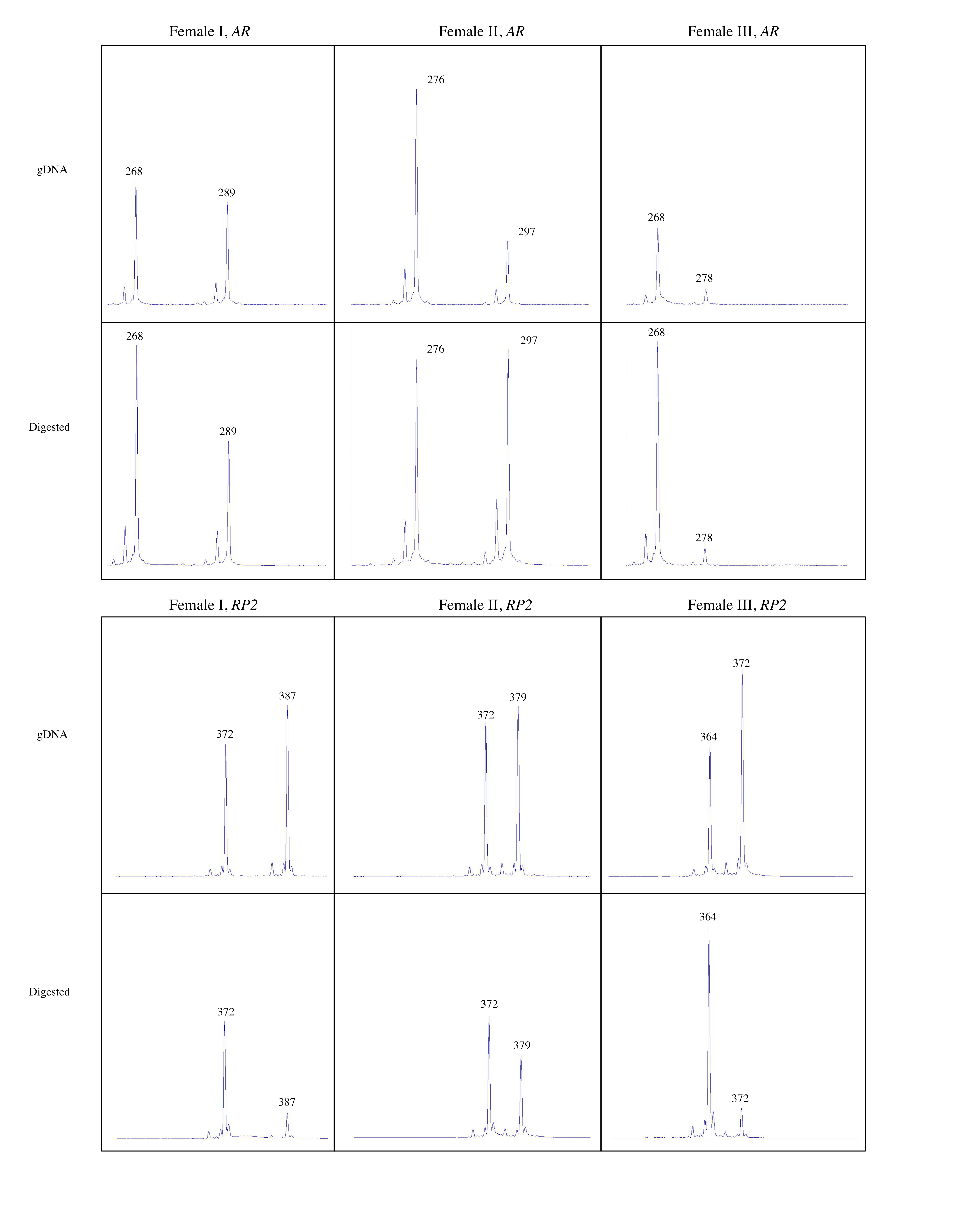

Supplement: Supplementary file 2 — Supplementary Figure 2. [file 41598_2023_34413_MOESM2_ESM.jpg]

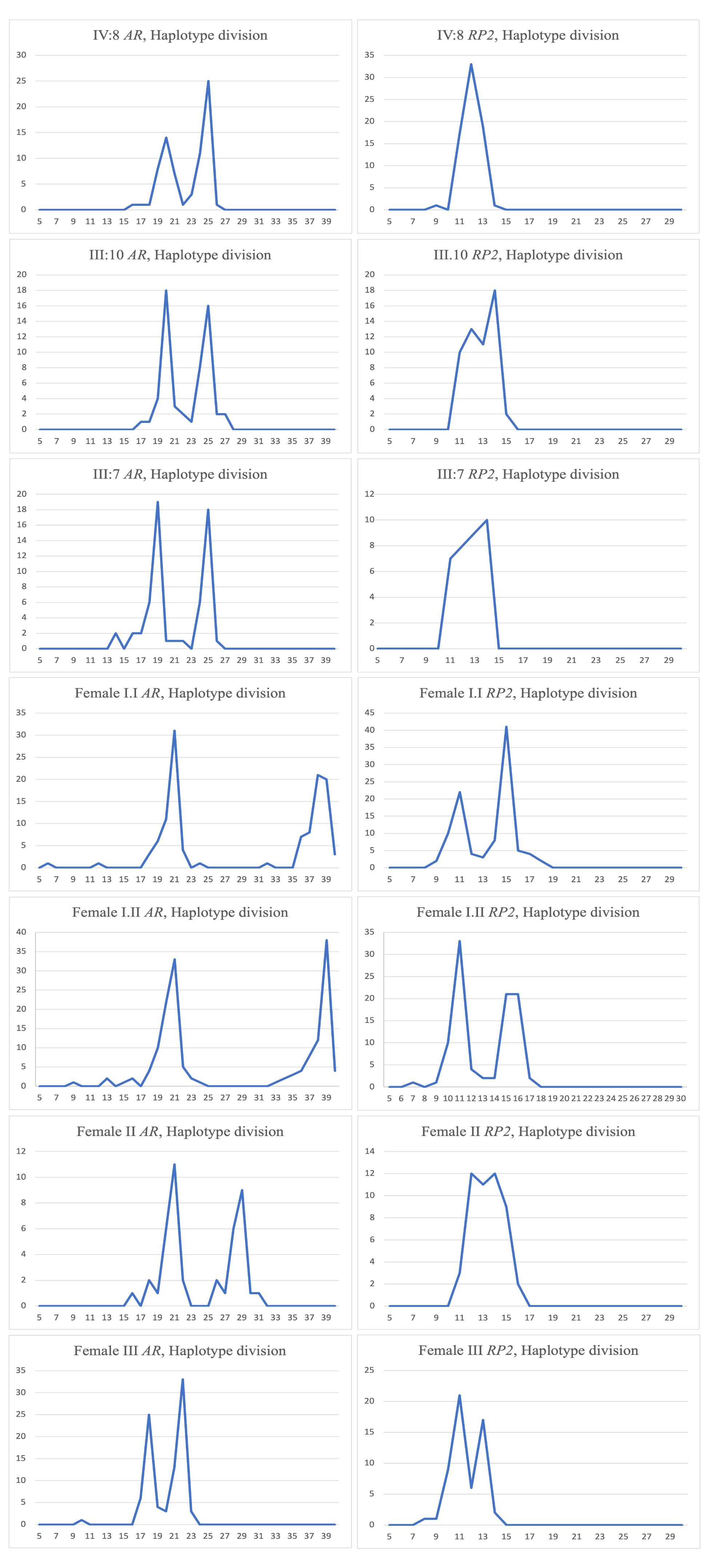

Supplement: Supplementary file 3 — Supplementary Figure 3. [file 41598_2023_34413_MOESM3_ESM.jpg]

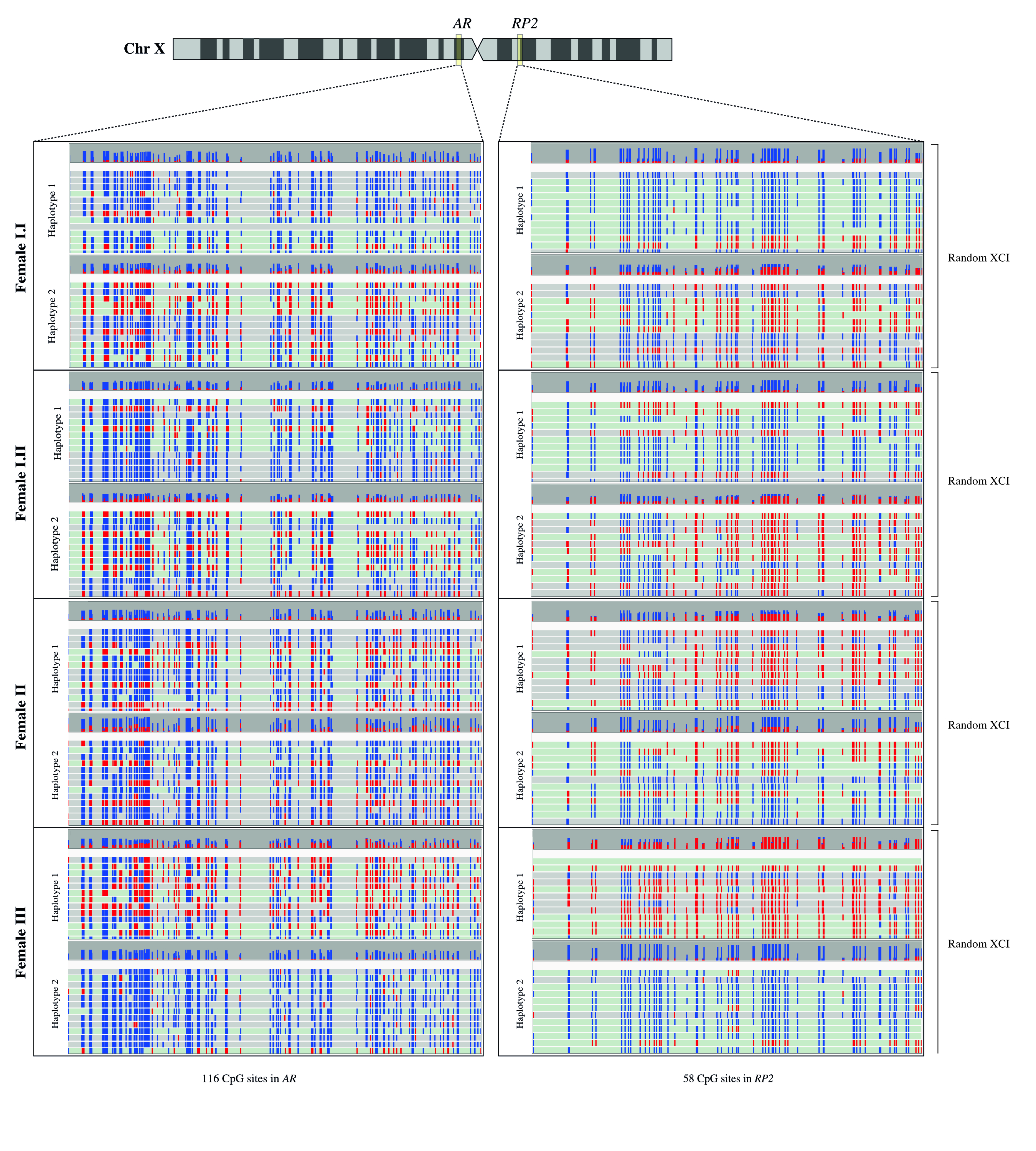

Supplement: Supplementary file 4 — Supplementary Figure 4. [file 41598_2023_34413_MOESM4_ESM.jpg]
