# Supplementary material for: A novel quantitative targeted analysis of X-chromosome inactivation (XCI) using nanopore sequencing
Source: Sci Rep. 2023 Aug 8;13:12856. doi: 10.1038/s41598-023-34413-3 (PMC10409790; doi:10.1038/s41598-023-34413-3)
Supplement: Supplementary file 6 — Supplementary Table 1. [file 41598_2023_34413_MOESM6_ESM.docx]

| **Target** | **gRNA sequence** | **Target position hg38** | **Target strand** |
| --- | --- | --- | --- |
| *AR* upstream | GGCCCGATCTATCCCTATGACGG | chrX:67543497 | + |
| *AR* upstream | CTACGCATTCTACTTACATATGG | chrX:67542472 | + |
| *AR* upstream | AGTATCTGCTGGCTTGGTCATGG | chrX:67543537 | + |
| *RP2* upstream | CAATGCTTATGTCCTCAACGAGG | chrX:46834684 | + |
| *RP2* upstream | CCATAGGCATCAGAAGGCCGAGG | chrX:46833596 | + |
| *RP2* upstream | TATGTTCAAATTAAGTCGGCCGG | chrX:46834073 | + |
| *AR* downstream | TCTGATGCAAACCTGAAGTAGGG | chrX:67547563 | - |
| *AR* downstream | GGCAATCTGAGTGTTCGCGCAGG | chrX:67546846 | - |
| *AR* downstream | TCCAGCTTGATGCGAGCGTGGGG | chrX:67546296 | - |
| *RP2* downstream | TCGAATCGATCATCTCCAACAGG | chrX:46837498 | - |
| *RP2* downstream | AAGAGAGTGCGGCAGCAGTTGGG | chrX:46837292 | - |
| *RP2* downstream | GGCACCAAAGAGATTTGCCGGGG | chrX:46837685 | - |
