# Supplementary material for: A novel quantitative targeted analysis of X-chromosome inactivation (XCI) using nanopore sequencing
Source: Sci Rep. 2023 Aug 8;13:12856. doi: 10.1038/s41598-023-34413-3 (PMC10409790; doi:10.1038/s41598-023-34413-3)
Supplement: Supplementary file 7 — Supplementary Table 2. [file 41598_2023_34413_MOESM7_ESM.docx]

| **Samples** | **Total nb of aligned reads** | **Nb of reads on-target for *AR*** | **Nb of reads on-target for *RP2*** | **Nb of reads on-target used in analysis for *AR*** | **Nb of reads on-target used in analysis for *RP2*** |
| --- | --- | --- | --- | --- | --- |
| IV:8 | 71480 | 73 | 71 | 58 | 37 |
| III:10 | 19871 | 58 | 54 | 46 | 43 |
| III:7 | 17674 | 59 | 34 | 59 | 25 |
| Female I.I | 44162 | 118 | 101 | 83 | 78 |
| Female I.II | 21393 | 155 | 97 | 105 | 85 |
| Female II | 15374 | 43 | 49 | 32 | 36 |
| Female III | 19396 | 88 | 57 | 77 | 49 |
