# Supplementary material for: A novel quantitative targeted analysis of X-chromosome inactivation (XCI) using nanopore sequencing
Source: Sci Rep. 2023 Aug 8;13:12856. doi: 10.1038/s41598-023-34413-3 (PMC10409790; doi:10.1038/s41598-023-34413-3)
Supplement: Supplementary file 9 — Supplementary Legends. [file 41598_2023_34413_MOESM9_ESM.docx]

**Supplementary information**

**Supplementary table 1:** All twelve guideRNAs (gRNAs) designed with the PAM sequence (green) and target sequence for Cas9 enrichment (black).

**Supplementary table 2:** Number of reads generated using the XCI-ONT analysis pipeline and number of reads used in the analysis.

**Supplementary table 3:** Calculated ratios of X-inactivation in all investigated individuals using the methylation frequency spanning 116 CpG sites in *AR* and 58 CpG sites in *RP2*.

**Supplementary figure 1:** Golden standard *RP2* analysis of two asymptomatic carrier females of X-linked intellectual disability (IV:8, III:10) and one none-carrier female of the same family (III:7). Numbers represents the fragment length observed before and after digestion using HpaII.

**Supplementary figure 2:** Golden standard AR and *RP2* analysis of three random females (Female I, II and III). Numbers represents the fragment length observed before and after digestion using HpaII.

**Supplementary figure 3:** Haplotype division using the repeats in the *AR* and *RP2* gene of all investigated individuals. On the x-axis is the number of repeats and on the y-axis is the number of reads called. IV:8 = *AR* repeat 19-20 (haplotype 1), 24-25 (haplotype 2) and *RP2* repeat 10-11 (haplotype 1), 13-14 (haplotype 2). III:10 = *AR* repeat 19-20 (haplotype 1), 24-25 (haplotype 2) and *RP2* repeat 11-12 (haplotype 1), 14-15 (haplotype 2). III:7 = *AR* repeat 18-19 (haplotype 1), 24-25 (haplotype 2) and *RP2* repeat 11-12 (haplotype 1), 14-15 (haplotype 2). Female I.I = AR repeat 20-21 (haplotype1), 38-39 (haplotype 2) and RP2 repeat 10-11 (haplotype 1), 15-16 (haplotype 2). Female I.II = AR repeat 20-21 (haplotype1), 38-39 (haplotype 2) and RP2 repeat 10-11 (haplotype 1), 15-16 (haplotype 2). Female II = AR repeat 20-21 (haplotype1), 28-29 (haplotype 2) and RP2 repeat 11-12 (haplotype 1), 13-14 (haplotype 2). Female III = AR repeat 17-18 (haplotype1), 21-22 (haplotype 2) and RP2 repeat 10-11 (haplotype 1), 13-14 (haplotype 2).

**Supplementary figure 4:** Visualization of XCI-ONT result using Integrative Genomics Viewer (IGV) presenting methylated CpG sites (red; Xi) and unmetylated CpG sites (blue; Xa) across the reads in the Androgen receptor (*AR*) and Retinis pigmentosa 2 (*RP2*) genes. Female I.I, I.II, II and III present different ratios of random XCI for both *AR* and *RP2*, except for Female III who present a skewed XCI ration for *RP2*. Top bar in each haplotype visualization indicates the read coverage (height) and the percentage of methylated and unmethylated calls at each position.

**Supplementary figure 5:** The methylation calling variability across all investigated CpG sites in the *AR* and *RP2* genes. X-axis: genomic start positions of 116 CpG sites for the *AR* gene and 58 CpG sites for the *RP2* gene. Y-axis: The Log-likelihood ratio scores (Log_Lik_Ratio) presenting the likelihood of a position being methylated or unmethylated. Methylated: Log_Lik_Ratio values >2.5. Unmethylated: Log_Lik_Ratio values <-2.5. Grey: Log_Lik_Ratio values <2,5 and >-2,5, where the methylation call did not meet the tresholds used for a true methylation call used in this study. Orange: Haplotype 1, based on repeat counts. Light blue: Haplotype 2, based on repeat counts. Each dot represents the Log_Lik_Ratio for each read at the position. A) Variability of all methylation calls for IV:8 for the *AR* gene. B) Variability of all methylation calls for IV:8 for the *RP2* gene. C) Variability of all methylation calls for III:10 for the *AR* gene. D) Variability of all methylation calls for III:10 for the *RP2* gene. E) Variability of all methylation calls for III:7 for the *AR* gene. F) Variability of all methylation calls for III:7 for the *RP2* gene. G) Variability of all methylation calls for Female I.I for the *AR* gene. H) Variability of all methylation calls for Female I.I for the *RP2* gene. I) Variability of all methylation calls for Female I.II for the *AR* gene. J) Variability of all methylation calls for Female I.II for the *RP2* gene. K) Variability of all methylation calls for Female II for the *AR* gene. L) Variability of all methylation calls for Female II for the *RP2* gene. M) Variability of all methylation calls for Female III for the *AR* gene. N) Variability of all methylation calls for Female III for the *RP2* gene. *The full span of the region used in the methylation call, and the number of CpG sites at each position can be found in Supplementary table 3.
